# Cyto-Histomorphological Analysis of Thyroid Lesions and Risk Assessment of Malignancy/Neoplasia: Insights From a North Indian Tertiary Oncology Center

**DOI:** 10.7759/cureus.82959

**Published:** 2025-04-24

**Authors:** Sadaf Haiyat, Zachariah Chowdhury, Paramita Rudra Pal, Shashikant Patne, Ipsita Dhal, Paramita Paul

**Affiliations:** 1 Department of Oncopathology, Homi Bhabha Cancer Hospital, Mahamana Pandit Madan Mohan Malviya Cancer Centre, Homi Bhabha National Institute, Varanasi, IND

**Keywords:** bethesda reporting system, fnac, histopathology, risk of malignancy, thyroid cytology

## Abstract

Background

Thyroid nodules, whether benign or malignant, are commonly identified as palpable or incidental findings. Accurate diagnosis is critical, with fine-needle aspiration cytology (FNAC) playing a crucial role in distinguishing between benign and malignant lesions. The Bethesda System for Reporting Thyroid Cytopathology (BSRTC) standardizes FNAC reporting and estimates the risk of malignancy (ROM), aiding treatment decisions. This study aims to determine the risk of malignancy for each category of the Bethesda System and to evaluate the sensitivity and specificity of FNAC in diagnosing thyroid swellings.

Methodology

Clinicopathological data of thyroid FNAC and corresponding thyroid resection cases, collected over four years at the Department of Oncopathology, Mahamana Pandit Madan Mohan Malviya Cancer Centre and Homi Bhabha National Institute, Varanasi, were analyzed.

Results

A total of 559 patients (372 females, 187 males) with a median age of 49 years were evaluated. Among the BSRTC categories, Category VI (32.4%) and Category II (29.2%) were the most common. ROM for each category was as follows: 50%, 25%, 30%, 85.71%, 97%, and 100%, respectively. FNAC demonstrated a sensitivity of 98%, a specificity of 64%, a positive predictive value of 96%, and a negative predictive value of 75%. Concordance between cytopathological and histopathological findings for malignant cases was 69.8%. Papillary thyroid carcinoma was the most common malignancy.

Conclusions

The ROM for categories I, II, and III was significant, highlighting the importance of the six-tier reporting system. The BSRTC system standardizes reporting and clinical management. Our data, primarily from an oncology center, may vary based on the expertise of the pathologist, laboratory setup, and patient demographics.

## Introduction

Thyroid nodules present a frequent diagnostic challenge in clinical settings, with both benign and malignant lesions often detected as palpable masses or incidental findings during routine examination. The prevalence of these nodules varies significantly, reported from 0.2-2% in pediatric populations to 4-10% in adults [1]. The increased use of ultrasonography has led to a marked increase in the identification of thyroid nodules [2]. Among these, it is estimated that 5-30% are ultimately diagnosed as malignant [3]. Distinguishing benign from malignant thyroid lesions is crucial for effective clinical management. Although initial suspicion may arise from clinical examination, confirmatory diagnostic methods, including radiological imaging and pathological assessment, are essential for establishing a definitive diagnosis.

Fine-needle aspiration cytology (FNAC) is a fundamental component of the diagnostic strategy for thyroid nodules. This minimally invasive procedure is widely employed because of its high diagnostic accuracy for differentiating between benign and malignant thyroid conditions [4]. The Bethesda System for Reporting Thyroid Cytopathology (BSRTC, 2007) has standardized FNAC reporting and assigned estimated risk of malignancy (ROM) to each diagnostic category [5]. These ROM estimates are vital for informing subsequent clinical management decisions and selecting appropriate treatment options.

Despite the advantages of FNAC, it has some limitations. Variability in interpretation among pathologists and differences in sample preparation techniques can lead to false-positive and false-negative results, thereby affecting the ROM associated with each Bethesda category. As a result, there is significant variability in ROM estimates across different institutions, particularly for BSRTC Categories III and IV. This highlights the need to generate institutional ROM data for each Bethesda category within specific clinical contexts. Such data are crucial for developing tailored management algorithms for thyroid nodules and assessing the effectiveness of routinely used diagnostic procedures in healthcare settings.

Establishing institutional ROM data not only aids in creating evidence-based management protocols but also allows institutions to benchmark their diagnostic practices against national and international standards. In addition, it serves as a foundation for ongoing quality improvement initiatives aimed at enhancing patient care and outcomes in the evaluation of thyroid nodules. The key objectives of this study are (1) to assess the risk of malignancy and neoplasia across the six BSRTC categories in a tertiary oncology center setting, and (2) to evaluate the sensitivity and specificity of FNAC in diagnosing thyroid swellings using histopathology as the gold standard.

## Materials and methods

This retrospective, observational study was conducted in the Department of Oncopathology at a tertiary cancer center in North India, following approval from the Institutional Ethics Committee, Mahamana Pandit Madan Mohan Malviya Cancer Centre (approval number: OIEC/11000657/2023/00002). This study included all patients whose thyroid FNAC and resection specimens were obtained from the Department of Pathology between January 2019 and April 2023.

The methodology involved retrieving all relevant thyroid FNAC and resection cases from the archives of the Department of Pathology. Smears were stained using Toluidine blue stain for rapid on-site evaluation of adequacy. Smears fixed in 95% ethanol were stained with Papanicolaou stain, and air-dried smears were stained with standard May-Grünwald-Giemsa stain for the final cytopathologic examination. Hematoxylin and eosin staining was also performed for FNAC smears.

Data were collected from electronic medical records, including patient demographics such as age and gender, presenting symptoms, and results from radiological investigations. Corresponding cytology smears and slides of excised specimens were also retrieved for a detailed review of cytopathological and histomorphological characteristics.

The collected data were systematically entered into the case study. Data analysis included a thorough discussion and interpretation of clinicopathological findings, as well as histopathologic and cytopathological features, highlighting diagnostic challenges and insights. The risks of malignancy (ROM) and neoplasia (RON) were calculated based on cases with histological follow-up. For RON estimation, both follicular adenoma and non-invasive follicular thyroid neoplasm with papillary-like nuclear features (NIFTP) were included alongside malignant neoplasms, whereas only malignant entities were considered for ROM.

All analyses were conducted using SPSS Software version 30 (IBM Corp., Armonk, NY, USA). The data were organized and analyzed using statistical methods to determine the distribution, including proportions, means, medians, and modes. The Kruskal-Wallis test compared median tumor sizes across cytodiagnosis categories.

## Results

**Figure 1:**
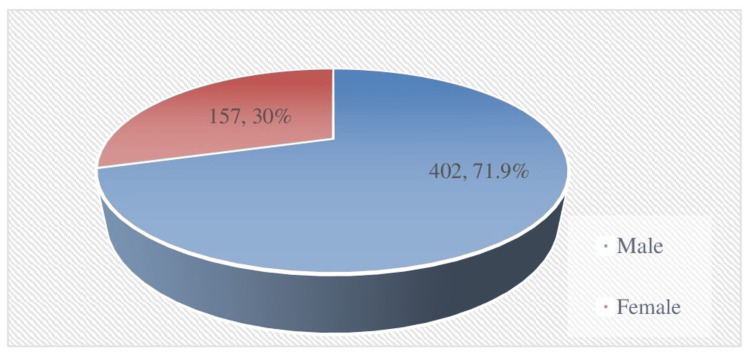
Gender distribution of 559 cases.

**Figure 2:**
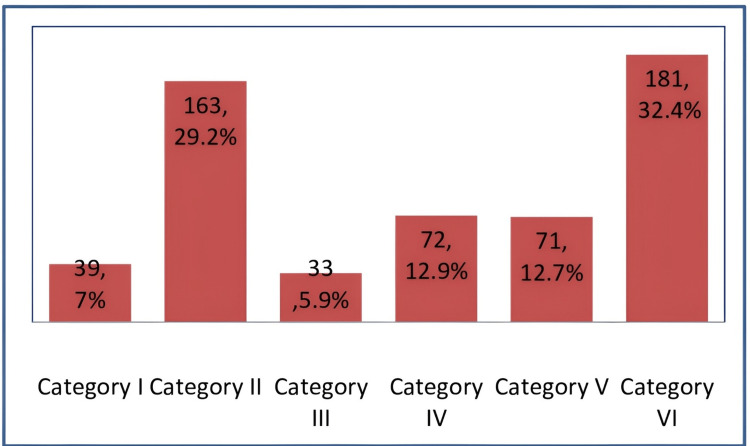
Frequency of different categories of thyroid cytology as per the Bethesda System for Reporting Thyroid Cytopathology (N = 559).

The study included 559 patients with a median age of 49 years (range = 14-85 years). The gender distribution showed a predominance of females (n = 372, 66.5%) compared to males (n = 157, 33.5%) (Figure 1). Patients were categorized into the following six BSRTC groups (2022) based on cytodiagnosis: 39 (7%) in Category I, 163 (29.2%) in Category II, 33 (5.9%) in Category III, 72 (12.9%) in Category IV, 71 (12.7%) in Category V, and 181 (32.4%) in Category VI (Figure 2).

Surgical procedures, including hemithyroidectomy (n = 93) and total thyroidectomy (n = 96), were performed in 189 patients, with 164 of the cases diagnosed as malignant (Table 1). Overall, 77 patients received radioactive iodine therapy.

**Table 1:** Different histopathological diagnoses of resected thyroid specimens across different thyroid Bethesda categories. BSRTC: Bethesda System for Reporting Thyroid Cytopathology; PTC: papillary thyroid carcinoma; Ca: carcinoma; FA: follicular adenoma; MNG: multinodular goiter; NIFTP: non-invasive follicular thyroid neoplasm with papillary-like nuclear features; WDTUMP: well-differentiated tumor of uncertain malignant potential; PD Ca: poorly differentiated carcinoma

Diagnostic BSRTC category	Cases with histopathology (n = 189)	Final diagnosis as malignant lesions (n = 164)	Final diagnosis as benign/low-risk neoplastic lesions
Non-diagnostic (Category I)	8	PTC (n = 1); medullary Ca (n = 1); follicular Ca (n = 2)	Colloid goiter (n = 3); FA (n = 1)
Benign (Category II)	12	PTC (n = 1); medullary Ca (n = 1); oncocytic Ca (n = 1)	MNG (n = 4); FA (n = 1); adenomatoid hyperplasia (n = 1); lymphocytic thyroiditis (n = 1); NIFTP (n = 2)
Atypia of undetermined significance (Category III)	10	PTC (n = 2); follicular Ca (n = 1)	MNG (n = 2); Hashimoto thyroiditis (n = 1); FA (n = 2); adenomatoid goiter (n = 1); NIFTP (n = 1)
Follicular neoplasm (Category IV)	28	Follicular Ca (n = 11); oncocytic Ca (n = 3); PTC (n = 4); FVPTC (n = 4); WDTUMP (n = 2)	FA (n = 3); NIFTP (n = 1)
Suspicious for malignancy (Category V)	34	Follicular Ca (n = 4); medullary Ca: (n = 3); FVPTC (n = 4); PTC (n = 18); PD Ca (n = 5); WDTUMP (n = 1)	Colloid nodule (n = 1)
Malignant (Category VI)	97	PTC (n = 42); oncocytic Ca (n = 1); medullary Ca (n = 5); poorly differentiated Ca (n = 19); FVPTC (n = 3)	

Among the 39 cases classified as non-diagnostic, eight patients with a high suspicion of malignancy underwent surgery, resulting in four (50%) cases confirmed as malignant, including papillary thyroid carcinoma (PTC), medullary carcinoma, and follicular carcinoma. The remaining cases were benign, including colloid goiters and follicular adenomas. In this category, RON was higher than ROM (62.5% vs. 50%) (Table 2).

**Table 2:** Risk of malignancy and risk of neoplasia for different thyroid Bethesda categories.

Thyroid Bethesda categories for cytology	Number of cases (%) (n = 559)	Mean age (years)	Cases with histopathology (n = 189)	Malignant histopathology	Risk of malignancy (%)	Risk of neoplasia (%)
Non-diagnostic	39 (7%)	48.20	8	4	50%	62.5%
Benign	163 (29.2%)	48.49	12	3	25%	50%
Atypia of undetermined significance	33 (5.9%)	45.30	10	3	30%	50%
Follicular neoplasm	72 (12.9%)	51.33	28	24	85.71%	100%
Suspicious For malignancy	71 (12.7%)	49.31	34	33	97%	97%
Malignant	181 (32.4%)	47.38	97	97	100%	100%

In the benign category (BSRTC Category II), 163 cases were identified, with three of 12 surgically removed nodules found to be malignant, yielding a malignancy rate of 25%. These included the follicular variant of PTC (FVPTC), medullary carcinoma, and Hurthle cell carcinoma, while the majority were benign, including multinodular goiter and follicular adenoma. Two cases were classified as NIFTP, resulting in a higher RON than ROM (50% vs. 25%) (Table 2).

For atypia of undetermined significance (AUS) category (BSRTC Category III), 10 of 33 cases underwent surgery, with three cases diagnosed as malignant (ROM = 30%). In the follicular neoplasm category (BSRTC Category IV), 28 of 72 cases had surgery, with 24 cases diagnosed as malignant (ROM = 85.71%), primarily consisting of follicular carcinoma and PTC, while three cases were identified as follicular adenoma and one as NIFTP. The RON for these categories was also higher than the ROM (50% vs. 30% and 100% vs. 85.7%, respectively) (Table 2).

**Figure 3:**
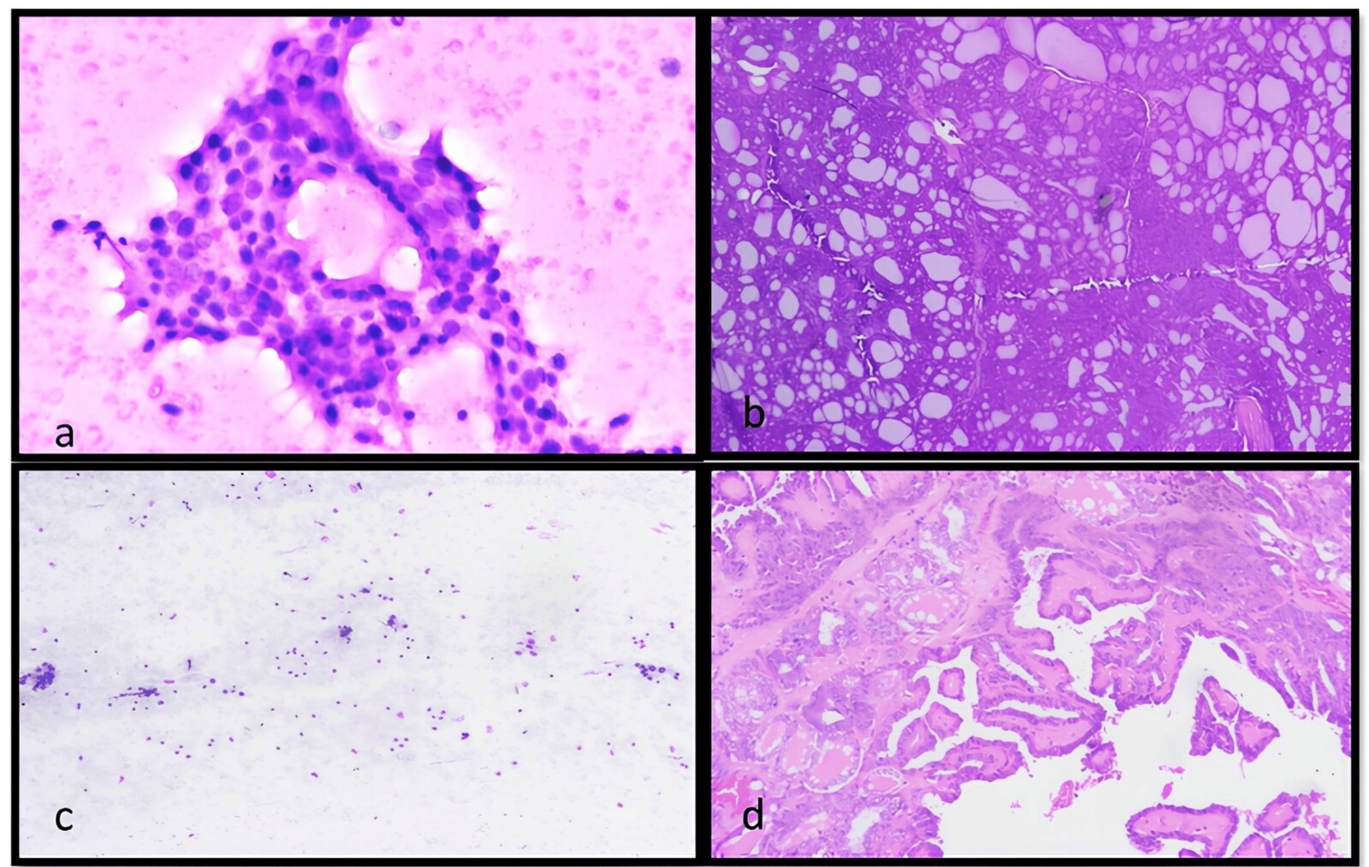
Photomicrograph of cytology and corresponding histopathology samples. False-positive case (a, b): Giemsa-stained smear diagnosed as suspicious of papillary thyroid carcinoma (40×) (a), but histological diagnosis was follicular nodular thyroid disease (b) (10×, H&E). False-negative case (c, d): Giemsa-stained smear diagnosed as colloid nodule (10×) (c), while histological diagnosis was papillary carcinoma of thyroid (d) (10×, H&E). This was misdiagnosed due to improper sampling of the tumor. H&E: hematoxylin and eosin

In the suspicious for malignancy (BSRTC Category IV) group, 34 of 71 patients underwent surgery, with 33 (97%) cases confirmed as malignant, including anaplastic thyroid carcinoma, PTC, and follicular carcinoma, while one case was a benign colloid nodule. In this false-positive case, cytodiagnosis of suspicious papillary carcinoma of the thyroid was made based on nuclear clearing, overcrowding, and focal nuclear grooving. Entrapment of cells in the blood was misinterpreted as nuclear overcrowding. The nuclear clearing that was observed was probably due to the staining issue (Figure 3). Among the 181 cases diagnosed as malignant cytology, surgery was performed in 97 cases, all of which were confirmed as malignant on histopathology (100%). RON and ROM for Categories V and VI were identical, as no NIFTP or follicular cases were found in these categories (Table 2).

In our study, in comparison with histopathology (n = 189), the FNAC procedure showed 154 true-positive cases, five false-positive cases, nine true-negative cases, and three false-negative cases. The five false-positive cases included four cases of Category IV and one case of Category V (Figure 3). All three false-negative cases were categorized as Category 2. The concordance between the cytopathological and histopathological findings of thyroid swelling was 91.22% based on FNAC and histopathological categorization of the lesion in BSRTC Categories II, IV, V, and VI cases. Categories I and III were excluded as these cases did not have a definitive FNAC diagnosis to compare with the histological diagnosis.

FNAC showed a sensitivity (true-positive rate) of 98% and specificity (true-negative rate) of 64%. The positive predictive value for suspicious and malignant cases was 96%, whereas the negative predictive value was 75%. Sensitivity and specificity were calculated, excluding BSRTC Categories III and I.

The median tumor size was 3 cm (range = 0.4 to 15 cm). The Kruskal-Wallis test indicated no significant difference in median tumor size across the different cytodiagnosis categories (p = 0.764).

Females were predominant across all age groups, particularly in the 21-45-year age group, which had the highest representation at 77.1%. Hormone status assessment showed that 94 (16.8%) patients had euthyroidism, 10 (1.8%) had hyperthyroidism, and 20 (3.6%) had hypothyroidism, with data unavailable for 435 patients.

A significant correlation was found between the Thyroid Imaging Reporting and Data System (TIRADS) and cytodiagnosis categories (p < 0.000), with higher TIRADS scores associated with a higher suspicion of malignancy. The benign cytodiagnosis categories (I and II) predominantly corresponded to lower TIRADS scores (TR2 and TR3), while more suspicious categories (III and IV) were spread across TIRADS scores TR3 to TR5. Notably, malignant cases (Category VI) were primarily associated with higher TIRADS scores (TR4, TR5, and TR6), supporting the use of TIRADS as a useful tool for evaluating the risk of malignancy in thyroid nodules (Table 3).

**Table 3:** Correlation of TIRADS score with Bethesda categories of thyroid cytology reporting. TIRADS: Thyroid Imaging Reporting and Data System

Cytodiagnosis category	TIRADS (n = 341)						
	TR2 (n = 17)	TR3 (n = 73)	TR4 (n = 124)	TR5 (n = 121)	TR6 (n = 3)	TR8 (n = 2)	TR9 (n = 1)
I	1 (5.9%)	8 (11%)	12 (9.7%)	4 (3.3%)	0 (0%)	0 (0%)	0 (0%)
II	10 (58.8%)	38 (52.1%)	35 (28.2%)	17 (14%)	0 (0%)	0 (0%)	0 (0%)
III	3 (17.6%)	4 (5.5%)	6 (4.8%)	3 (2.5%)	0 (0%)	0 (0%)	0 (0%)
IV	2 (11.8%)	10 (13.7%)	18 (14.5%)	21 (17.4%)	1 (33.3%)	0 (0%)	0 (0%)
V	0 (0%)	7 (9.6%)	18 (14.5%)	19 (15.7%)	1 (33.3%)	0 (0%)	0 (0%)
VI	1 (5.9%)	6 (8.2%)	35 (28.2%)	57 (47.1%)	1 (33.3%)	2 (100%)	1 (100%)

## Discussion

Our study, which included 559 patients, adds to the expanding research on the diagnostic value of FNAC in the evaluation of thyroid nodules. The median age of the patients was 49 years, with a predominance of females, a trend consistent with that of earlier studies [6,7]. This gender distribution aligns with the widely recognized higher incidence of thyroid disorders among women [7].

BSRTC provides a standardized framework for categorizing thyroid nodules based on cytological findings. Using a uniform six-tier system with defined diagnostic categories, risk predictions for malignancy, and management recommendations, BSRTC reduces the occurrence of inconclusive cases, ensures consistency across laboratories, and aids clinicians in determining the appropriate treatments [8-10].

In our cohort, Category VI (malignant) was the most common, followed by Category II (benign), a pattern similar to that in other studies performed in cancer care centers [11].

Non-diagnostic FNAC cases accounted for 7% in our study, a rate lower than that reported in other studies at 11.1-18.6% [8,11-13]. However, ROM in our non-diagnostic cases was notably high (50%), exceeding the BSRTC benchmark of 5-10%. Our data also showed a lower proportion of benign cases (29.6%) compared to the 61.3-73.8% reported in similar studies by Tepeoglu et al. and Bhasin et al. [8,12,14]. This disparity may reflect the referral bias in our tertiary cancer center, where most patients present with a high suspicion of malignancy. As a tertiary cancer care center, only selected cases with a non-diagnostic or benign FNAC diagnosis underwent surgery if there was clinical suspicion of malignancy, based on specific clinical and radiological factors (e.g., lesion size >3 cm, compression of surrounding structures, or cosmetic concerns). Other cases were referred to non-oncology centers for further follow-up.

False-negative results in these two categories were primarily due to non-representative material on cytology slides, emphasizing the importance of clinical judgment in managing lesions concerning clinical and radiological features.

The AUS (BSRTC III) category remains one of the most contentious categories. This category includes smears with cytological and architectural atypia that are not classified as follicular neoplasms [8]. The ROM for AUS is typically between 10% to 30% [8,9,15]. Variability in AUS incidence (0.7-18%) and malignancy rates (6-48%) in resected cases has been noted in various studies [16,17].

Our study reported 9% of cases as AUS, comparable to the rates of 9.8-11% observed in other studies [8,11,12,14], although lower rates (3%) have also been documented [9]. The ROM for AUS in our cohort was 30%, consistent with the broader range of 12.7-37.8% reported in other studies [16,17,18-23], although higher rates, such as 52.9% reported by Kamboj et al., have also been noted (Table 4). Low cellularity and hemorrhagic smears obscuring nuclear features were common reasons for missing a diagnosis of malignancy in AUS cases in our study. In our study, the primary factor contributing to the relatively high ROM for Category III was selection bias related to surgical decisions at our institution. For BSRTC Category III cases, the decision to proceed with surgery (mainly hemithyroidectomy) was based on clinical and radiological indications of malignancy. Ancillary testing for additional risk stratification was not utilized for Category III cases included in our study, as such resources were unavailable at our center.

**Table 4:** Comparison of our study results with other studies published in the literature. BSRTC: Bethesda System for Reporting Thyroid Cytopathology; ROM: risk of malignancy

Study	Total cases	Year	Non-diagnostic		Benign		Atypia of undetermined significance		Follicular neoplasm		Suspicious for malignancy		Malignant	
			Cases	ROM	Cases	ROM	Cases	ROM	Cases	ROM	Cases	ROM	Cases	ROM
BSRTC, 2022 [4]	-	-	-	5–20%	-	2–7%	-	13–30%	-	23–34%	-	67–83%	-	97–100%
Kamboj et al. [11]	431	2019	18.6%	78.6% (11/14)	30.2%	27.7% (5/18)	10.4%	52.9% (9/17)	6.3%	53.8% (7/13)	7.9%	95% (19/20)	26.7%	96.7% (58/60)
Jo et al. [20]	3,080	2010	18.6%	8.9% (12/135)	59%	1.1% (20/1792)	3.4%	17% (9/53)	9.7%	25.4% (45/177)	2.3%	70% (39/56)	7%	98.1% (151/154)
Mahajan et al. [24]	4,562	2017	3.5%	50% (2/4)	79.6%	7.8% (13/166)	2.5%	50% (6/12)	3.9%	23.6% (9/38)	0.5%	75% (3/4)	9.8%	85.4% (94/110)
Present study	559	2023	7%	50% (4/8)	29.2%	25% (3/12)	5.9%	30% (3/10)	12.9%	85.71% (24/28)	12.7%	97% (33/34)	32.4%	100% (97/97)

Follicular neoplasm (BSRTC IV) was identified in 12.9% of cases in our study, higher than that reported by Theoharis et al., Kamboj et al., Mufti et al., and Mahajan et al. [11,13,23,24]. Follicular neoplasm cases in our cohort had an ROM of 85.7%, substantially exceeding the 12.7-36% range reported elsewhere [12,23,25,26].

The suspicious for malignancy BSRTC V) category, with an expected ROM of 50-75%, guides patients toward surgical management [15]. In our cohort, 12.7% of cases were classified as suspicious for malignancy, higher than the 1.3-7.9% reported in other studies [8,11-13]. The ROM in our study was 97%, slightly exceeding rates of 70-95% reported previously [11,12,19,20].

Cases classified as malignant (BSRTC VI) typically constitute 4-8% of thyroid FNACs, with total thyroidectomy as the standard treatment [12]. As a tertiary care center for oncology, we also receive cases referred from peripheral regions, leading to a higher incidence of malignant cytology (32.4%), which is significantly higher than the 2-5.2% reported in other studies [3,8,9], but similar to Kamboj et al. [11]. The ROM for malignant cases was 100%, consistent with BSRTC recommendations and previous studies reporting rates of 98-100% [12,19,20].

In our study, only 52% of cases (131 out of 252) classified as BSRTC Categories V and VI underwent surgery during the study period. In some instances, only cytology slides were submitted for review. Additionally, 17 cases were lost to follow-up after FNAC.

The diagnostic performance of FNAC in our study aligns with that reported in previous studies. The sensitivity was 98% and the specificity was 64%, highlighting its effectiveness in distinguishing malignant and benign nodules [11,24]. The positive and negative predictive values for suspicious and malignant cases were 96% and 75%, respectively, reinforcing the role of FNAC in clinical decision-making [7,20].

Our study evaluated RON by incorporating NIFTP, classified as low risk in the 2022 WHO guidelines. Benign cases in our study showed a higher RON (41.6%) than that reported by Mahajan et al., but lower than that reported by Kamboj et al. The atypia, follicular neoplasm, and suspicious for malignancy cases in our study had a high RON (40%, 89.2%, and 97%, respectively) (Table 5). Inclusion of NIFTP increased the RON for Categories II, III, and IV. Excluding NIFTP decreased ROM in Category II cases more significantly than that described in BSRTC [4].

**Table 5:** Comparison of our study results with other studies published in the literature. RON: risk of neoplasia

Study	Total cases	Year	Non-diagnostic		Benign		Atypia of undetermined significance		Follicular neoplasm		Suspicious for malignancy		Malignant	
			Cases	RON	Cases	RON	Cases	RON	Cases	RON	Cases	RON	Cases	RON
Kamboj et al. [11]	431	2019	18.6%	78.6% (11/14)	30.2%	27.7% (5/18)	10.4%	52.9% (9/17)	6.3%	53.8% (7/13)	7.9%	95% (19/20)	26.7%	96.7% (58/60)
Mahajan et al. [24]	4,562	2017	3.5%	75% (3/4)	79.6%	11.3% (19/166)	2.5%	66% (8/12)	3.9%	65.7% (25/38)	0.5%	100% (4/4)	9.8%	88.1% (97/110)
Present study	559	2023	7%	50% (4/8)	29.2%	41.6% (5/12)	5.9%	40% (4/10)	12.9%	89.2% (25/28)	12.7%	97% (33/34)	32.4%	100% (97/97)

Although FNAC remains a critical diagnostic tool, its limitations include sampling errors and indeterminate results, necessitating supplementary diagnostic techniques and clinical correlation [11,27]. Combining FNAC results with clinical and radiological data optimizes care and reduces unnecessary surgery.

Surgical resection continues to be the definitive method for the diagnosis and treatment of thyroid malignancies. The strong concordance (91.2%) between FNAC and histopathological findings in our study highlights the value of FNAC in preoperative risk stratification and treatment planning [28,29].

A key limitation of our study was the small number of surgical resection specimens and the selection bias in choosing cases for surgery. As a result, the risk of malignancy for Categories I, II, and IV was higher than that reported in BSRTC 2002. A longer study period might have led to an increased number of surgical resections. Additionally, following up on cases referred to other centers could have provided a more accurate ROM, particularly for Categories I and II.

## Conclusions

This study provides ROM and RON estimates for each Bethesda system category, with Categories VI (malignant) and II (benign) revealing the highest and lowest ROM, respectively. Although FNAC is a reliable method for diagnosing thyroid nodules, its specificity and negative predictive value are relatively low, suggesting the risk of false positives and negatives. The concordance between FNAC and histopathology was high, highlighting FNAC as a useful, although not definitive, diagnostic tool, particularly when confirming malignancy.
